# Simulated In Vitro Digestive Characteristics of Raw Yam Tubers in Japanese Diet: Changes in Protein Profile, Starch Digestibility, Antioxidant Capacity and Microstructure

**DOI:** 10.3390/foods11233892

**Published:** 2022-12-02

**Authors:** Chuang Zhang, Sunantha Ketnawa, Sukanya Thuengtung, Yidi Cai, Wei Qin, Yukiharu Ogawa

**Affiliations:** 1 College of Food Science and Technology, Nanjing Agricultural University, Nanjing 210095, China; 2 Graduate School of Horticulture, Chiba University, Chiba 271-8510, Japan

**Keywords:** raw yam, *tororo*, in vitro digestion, digestive characteristics

## Abstract

The consumption of raw yam tuber through grated yam “*tororo*” is a major and popular diet in Japan. However, few studies have been undertaken to evaluate the digestive characteristics of raw yam tubers. This study aimed to fill this gap by investigating the changes in the protein profile, protein and starch digestibility, antioxidant capacity and microstructure of two typical yam tubers (Nagaimo N-10 and Nebaristar) in the Japanese diet, applying a simulated in vitro digestion method. Results showed that both samples contained a considerable protein content of about 11% (dry basis) and a protein digestibility of 43–49%. The electrophoretic patterns confirmed that dioscorin was the main protein of the yam tuber, and it could be digested into peptides and free amino acids with low molecular weight during in vitro digestion. The starch hydrolysis results suggested that eating raw yam tuber cannot induce a fast glycemic increase for consumers due to a low starch digestibility of 4.4–6.1%. In addition, Nebaristar showed a higher bioaccessibility in some key amino acids and total phenolic content than the Nagaimo N-10. This study provides some essential nutritional information and simulated digestion behaviours of the raw yam tubers, which could be useful for consumers and industries when buying and processing yam tubers from the perspective of changes in the nutritional profile during digestion.

## 1. Introduction

Yam (*Dioscorea* spp.) is a member of the monocotyledonous family that is widely consumed in east Asian countries such as China and Japan. In 2020, the Food and Agricultural Organization (FAO) of the United Nations reported that the global production of yam was about 74.8 million tons [1]. Yam is considered to be a great and nutritional plant food to diversify crops in order to address hunger and malnutrition [2]. The rhizome of *Dioscorea opposita* has been used in Chinese herbal medicine and listed in the pharmacopoeia of China. Yam has gained much attention in research owing to the presence of bioactive compounds such as dioscorin, dioscin, phenolics, flavonoids and tannins. The consumption of yam has been associated with a variety of health benefits due to its antioxidant, anti-inflammatory, immunomodulatory and anticancer properties [3].

In China, yam tubers are normally consumed in the thermal cooked form, such as steaming and boiling, whereas the Japanese generally consume yam tubers through grated yam “*tororo*” that is the main art of cooking in Japan [4]. Yam tuber contains a mass of mucilage, contributing to the high viscosity and special taste of *tororo*. It is known that the viscosity of yam tuber mucilage is mainly due to the viscous storage protein, which was identified as dioscorin [5,6]. Silva do Nascimento, Caju de Oliveira [7] studied yam protein digestibility and the peptide profile using simulated in vitro digestion, providing information for understanding the potential bioactive activity of the generated small molecules for human health.

Over the last decades, studies have reported the nutritional values of different *Dioscorea* species, e.g., Chinese yam tubers (*Dioscorea opposita*), containing ~65% starch, ~9% protein and ~1.2% fibre. They are also a rich source of minerals and phytochemicals such as dioscin and allantoin [8]. As a high-nutrition plant food, eating raw yam tuber is popular in the Japanese diet. Although a few studies have reported changes in the profile of the key viscous protein dioscorin during digestion, to the best of our knowledge, the digestive characteristics of the protein are still unknown, as well as those of the starch, antioxidants and microstructure of the raw yams consumed as *tororo* in the Japanese diet.

As mentioned above, consuming *tororo* is popular and deemed to be part of a healthy diet in Japan. Therefore, this study aimed to evaluate the simulated in vitro digestive characteristics of raw yam tuber by investigating the changes in protein profile, starch digestibility, antioxidant capacity and microstructure, using two typical yam tubers in different varieties (Nagaimo N-10 and Nebaristar) in Japan as samples. The findings provide fundamental information on the simulated in vitro digestive behaviours and nutritional bioaccessibility of the raw yam tuber to fill the research gaps in “*tororo*” in the Japanese diet, offering a reference to consumers when selecting yam tubers for being eaten raw and food industrial development of yam tubers from a nutritional perspective.

## 2. Materials and Methods

### 2.1. Materials and Reagents

**Figure A1:**
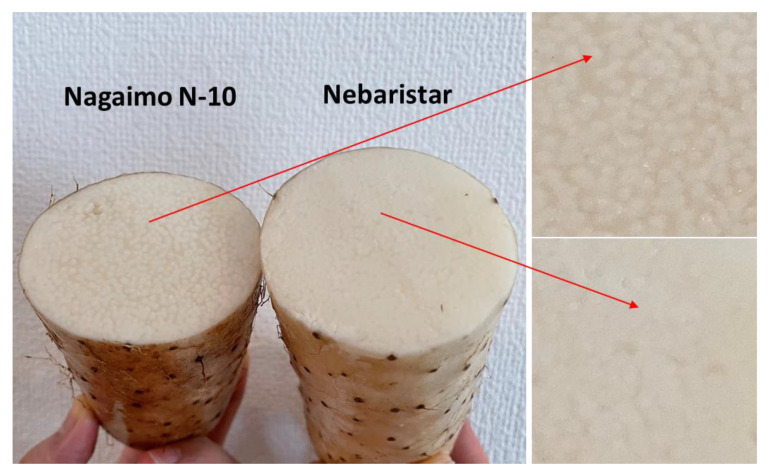
The appearance of the yam tuber samples.

Yam tubers Nagaimo N-10 (*Dioscorea polystachya Turcz.*) and Nebaristar (*Dioscorea polystachya*, cv. Nebaristar) (refer to Figure A1) were purchased from the local supermarket in Matsudo city, Chiba prefecture, Japan. Pepsin and pancreatin from porcine sources, invertase from baker’s yeast, gallic acid, Folin–Ciocalteu reagents and the standard (±)-6-Hydroxy-2,5,7,8tetramethylchromane-2-carboxylic acid (Trolox) were all purchased from Sigma-Aldrich (St. Louis, MO, USA). Amyloglucosidase (AMG) was purchased from Megazyme International Ireland Ltd. (Wicklow, Ireland). ABTS diammonium salt and all other chemicals of analytical grade were purchased from Wako Pure Chemical Corporation (Tokyo, Japan). Ultrapure water was used as needed.

### 2.2. Samples Preparation

Washed and peeled fresh yam tubers were cut into small pieces and then blended with distilled water (yam/water ratio of 1:1, *w*/*w*) for 2 min using a blender (THM310, Tescom, Tokyo, Japan). The obtained mixtures were then used for the in vitro digestion experiment.

### 2.3. Moisture Content

The peeled yam tuber was cut into small pieces (3 × 3 × 3 mm), and the moisture content was determined gravimetrically by drying in an oven (WFO-400, Tokyo Rikakikai, Tokyo, Japan) at 105 °C for 24 h [9].

### 2.4. Simulated In Vitro Gastrointestinal Digestion

A simulated two-stage in vitro gastrointestinal digestion was conducted according to Tamura, Singh [10] and Zhang, Quek [11] with modifications. Simulated gastric fluid (SGF) (100 mL) was made up of 0.48 g pepsin and gastric fluid buffer (adjust to a final volume of 100 mL). Simulated intestinal fluid (SIF) (100 mL) consisted of 0.4 g of pancreatine, 0.03 g of invertase, 8 mL of AMG and intestinal fluid buffer (adjust to a final volume of 100 mL). For the gastric fluid, buffer (1 L) was prepared by dissolving 2 g of NaCl and 7 mL of 12 mol/L HCl in 900 mL distilled water, followed by adjusting pH to 2.0 and filling up to a final volume of 1 L. The intestinal fluid buffer (1 L) was made by dissolving 6.8 g of KH_2_PO_4_ and 77 mL of 0.2 M NaOH in 750 mL distilled water, followed by adjusting pH to 6.8 and adding distilled water up to a final volume of 1 L.

The blended yam mixture (140 g) was transferred into the jacketed glass reactor and stirred at 500 rpm using a stir bar throughout the simulated digestion. The SGF (19 mL) was then added into the reactor, and the pH was re-adjusted to 2.0. An assistance of manual agitating (5 times/min) by a stir stick was needed for 10 min after the digestion commencement due to the high viscosity of the mixture. The simulated gastric digestion (SGD) was performed in the reactor at 37 °C for 120 min. Subsequently, the pH was re-adjusted to 6.8, and 23 mL of SIF was added to the gastric chyme. The simulated intestinal digestion (SID) was conducted with similar incubation conditions for another 120 min. The pH at each digestion stage was continuously maintained by addition of 6 mol/L HCl or 1 mol/L NaOH. Samples collections were completed at the initial time of SGD after pH adjustment, 60 and 120 min after SGD, the initial time of SID after pH adjustment and 60 and 120 min after SID, and the samples were referred to as G0, G60, G120, I0, I60 and I120, respectively. The samples were used for the analyses as described in Section 2.6, Section 2.7, Section 2.8, Section 2.9 and Section 2.10. The in vitro digestion experiments were performed in triplicate.

### 2.5. Starch Hydrolysis

To examine the starch digestibility of the yam samples, simulated in vitro digestion was performed in an individual tube for each sampling time point, under similar reaction conditions as described in Section 2.4. Briefly, a mixture (26 mL) consisting of yam slurry (10 mL) and 0.1 M gastric fluid buffer (16 mL) was prepared, and 2.5 mL of the mixture was mixed with 2.5 mL of SGF for incubation in a shaking water bath (T-25, Thomas Kagaku, Tokyo, Japan) at 37 °C, 70 strokes/min for 120 min. Then, 5 mL of SIF was individually added into the simulated gastric chyme for SID at similar incubation conditions for another 120 min. The samples were collected at 0, 60 and 120 min after SGD and 5, 20, 30, 60, 90 and 120 min after SID, which were referred to as G0, G30, G60, I5, I20, I30, I60, I90 and I120. Subsequently, 0.5 mL of each collected supernatant at each time point was immediately mixed with 2.5 mL of 95% ethanol to terminate the enzymatic reaction. The ethanol mixed solution was centrifuged at 2000× *g* for 10 min, and 0.2 mL supernatant was then incubated with 0.4 mL AMG/invertase solution at 37 °C for 10 min to convert all the potential oligosaccharides and disaccharides into glucose. The AMG/invertase solution (10 mL) was made up of AMG (0.2 mL), invertase (7.5 mg) and 9.8 mL potassium acetate buffer (pH 5.2).

The glucose concentration of the sample was determined according to the D-glucose assay kit (GOPOD Format K-GLUC 02/18, Megazyme International Ireland Ltd.). The results were represented as percentage of starch hydrolysis:

(1) $$S_{H\;}\left(\%\right)=0{.9\;\times \;G}_{p}/S_{i}$$

where *S*_H_ (%) refers to percentage of starch hydrolysis, *G*_p_ represents the amount of glucose produced, and *S*_i_ is the initial amount of total starch. The conversion factor of 0.9 was calculated from the molecular weight (MW) of starch monomer divided by the MW of glucose (162/180 = 0.9).

### 2.6. Total Protein Content and Protein Digestibility

The total soluble nitrogen contents of the yam samples were determined by a CN coder (MT-700 Mark 2, Yanaco, Tokyo, Japan) based on the Dumas method principle using hippuric acid as the standard [12], and the protein content of the supernatants obtained at different stages during in vitro digestion was determined according to the Pierce^TM^ BCA protein assay kit (NO. 23227, Thermo Scientific, Rockford, IL, USA). The protein contents of samples were calculated by the following equation:

Protein content (%) = nitrogen (%) × factor (6.25)(2)

The protein digestibility was expressed as follows:

Protein digestibility (%) = B/A × 100%(3)

where A is the total protein content of the yam sample, and B is the protein content of the supernatant digestion fluid at different digestion stages.

### 2.7. Soluble Protein Fractions and Distribution

The protein patterns of samples were determined via the sodium dodecyl sulfate polyacrylamide gel electrophoresis (SDS-PAGE), using a NuPAGE^TM^ Bis-Tris gradient precast gel (4–12% gradient) in a Novex XCell Mini-Cell (Invitrogen, Thermo Scientific Ltd.). In brief, 65 μL of sample was mixed with 25 μL of NuPAGE^TM^ LDS sample buffer and 10 μL of NuPAGE^TM^ to achieve a total volume of 100 μL, followed by an incubation at 70 °C for 10 min. Then, each sample mixture containing 20 mg protein with the different volumes calculated according to the previous BCA analysis was loaded into the gel, and the protein standard marker (Thermo Scientific) was used as the reference. The electrophoresis was run at a constant voltage of 200 V for 45 min. Finally, the gel was stained with SimplyBlue™ SafeStain (Thermo Scientific) overnight, followed by a de-staining treatment using distilled water until the background of the gel was clear.

### 2.8. Antioxidant Capacity

The antioxidant capacity of the yam digestion fluid at different digestion stages was determined by the ABTS assay according to Zhang, Khoo [13]. In brief, ABTS solution (7 mM, in 20 mM acetate buffer, pH 4.5) was mixed with K_2_S_2_O_8_ (2.45 mM) with a ratio of 1:1. The mixture was then left in a dark place for 12 to 16 h. Then, it was diluted by the acetate buffer to obtain an absorbance of 0.70 ± 0.01 at 740 nm. Thereafter, each sample was appropriately diluted, and an aliquot of 10 μL was added into a 96-well plate followed by 190 μL of the diluted ABTS solution. The mixture was left to react for 60 min in a dark place, and the absorbance was then measured using a microplate reader (Multiskan FC, Thermo Fisher Scientific, Waltham, MA, USA) at 740 nm. Trolox solutions with different concentrations (0.4, 0.3, 0.2, 0.1, 0.05 and 0 mM) were made in ethanol and used to establish a standard curve (R^2^ > 0.99) for this assay.

### 2.9. Total Phenolic Content (TPC)

The TPC of the digestion fluid at different digestion stages was determined using the Folin–Ciocalteu (FC) assay [14] with minor modification. Briefly, 25 μL of each appropriately diluted sample was mixed with 125 μL of 10-fold diluted FC reagent in a 96-well plate to react for 10 min. Then, 125 μL of Na_2_CO_3_ (7.5%, *w*/*v*) was added to each of the mixture to react for 60 min prior to the absorbance being measured at 740 nm. Gallic acid water solutions with different concentrations (0.6, 0.4, 0.2, 0.1, 0.05 and 0 mM) were prepared and used to establish a standard curve for this assay (R^2^ > 0.99). The TPC was expressed as mg gallic acid equivalent (GE) per mL of the digestion fluid. The FC assay is only an approximate method for TPC determination because of a large number of components in the sample that interfere with the assay [15].

### 2.10. Free Amino Acids

The free amino acid content was determined using an automatic amino acid analyser (JLC-500/V2, Jeol, Tokyo, Japan) according to the post-label ninhydrin method. Briefly, the collected supernatants were appropriately diluted, and the pH values were adjusted to the range of 2 to 3, followed by filtering using 0.45 μm filters. Then, the filtered supernatants were subjected to the amino acid analyser. The separation of free amino acids was performed using the cation exchange resin in a high separation mode with a lithium citrate buffer system. Then, the separated free amino acids were derivatized with the ninhydrin reagent and detected with a visible light detector. The open Type AN-II and Type B (Wako Pure Chemical, Osaka, Japan) were used as the standards.

### 2.11. Morphological Observation

The yam samples were carefully cut into small pieces (3 × 3 × 1 mm) and digested under similar conditions as described in Section 2.4. The yam pieces before digestion (G0) and the digested samples at G60, G120, I60 and I120 were collected and immediately stored at −80 °C until the freeze drying of samples was performed. Then, the freeze-dried samples were sputter-coated with gold (Ion sputter JFC-1100, Jeol, Tokyo, Japan), and the microstructures were observed using scanning electron microscope (SEM) (SU1510, Hitachi, Tokyo, Japan) at an accelerating voltage of 5 kV.

### 2.12. Statistical Analysis

All experiments and analyses were done in triplicate. A one-way ANOVA and Duncan’s test were performed using SPSS 23 (IBM, New York, NY, USA) to study the statistical differences of the mean values. A significant difference between samples is considered as *p* < 0.05.

## 3. Results and Discussion

### 3.1. Starch Digestibility

**Figure 1:**
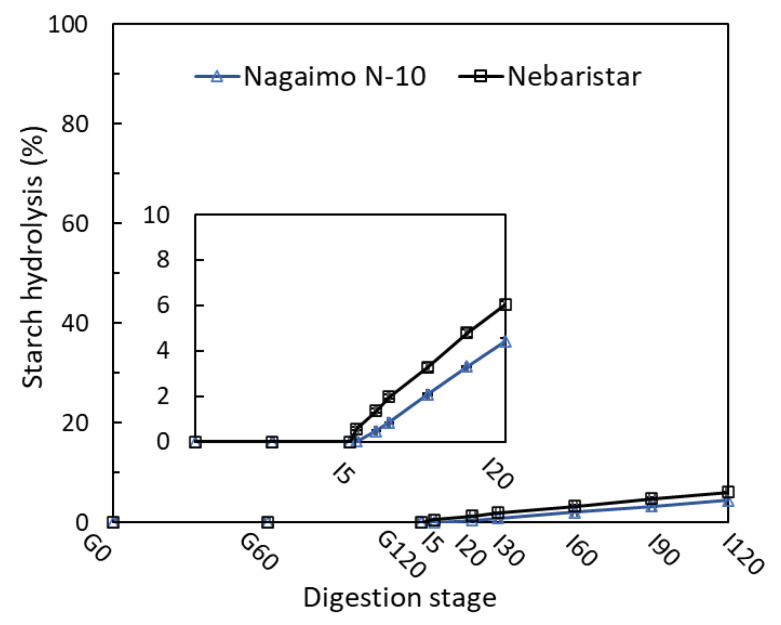
Changes in starch hydrolysis (%) of the raw yam tubers during simulated in vitro digestion.

Starch is a major source of carbohydrates consumed by human bodies, which is also a primary component in yam tubers. The samples of Nagaimo N-10 and Nebaristar showed a low percentage in starch hydrolysis (%, SH) during the simulated in vitro digestion of 4 h (Figure 1). In the SGD process, the SH of yam samples was not observed due to the absence of amylases in the SGF. However, it was increased after the SI digestion started, reaching 4.4% and 6.1% for Nagaimo N-10 and Nebaristar after 2 h incubation, respectively. In contrast, the SH of the yam starch in boiled, steamed and microwaved yam tubers after 4 h of in vitro digestion were relatively high (>78%) (data not shown). Similar results were mentioned by Guo, Yu [16], who found that the starch in raw wheat flour was hydrolysed very slowly, with only about 20% starch hydrolysis after 2 h of simulated in vitro digestion. However, wheat starch samples treated by heating with 20–70% water for 5–20 min showed a very high digestion percentage of >80% under similar incubation conditions.

Starch hydrolysis is closely related to its gelatinization, and a higher degree of gelatinization had a positive effect on SH [17]. Starch structural disruption would occur under heat treatments such as boiling, steaming and microwaving, leading to a high SH. The results found in the current study suggested that raw yam tubers would not induce a fast glycemic increase for consumers, although they contain a high total starch content of 67.3% for Nagaimo N-10 and 72.4% for Nebaristar (Table A1). This also indicates that eating raw yam could be beneficial for consumers who have requirements for their body’s blood sugar control.

### 3.2. Protein Digestion

#### 3.2.1. Protein Digestibility

**Figure 2:**
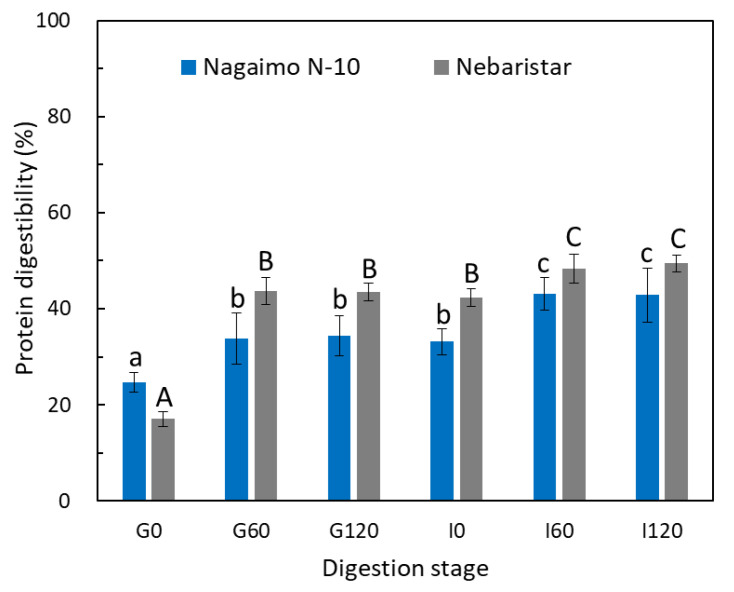
Changes in protein digestibility (%) of the raw yam tubers during simulated in vitro digestion. Different lowercase letters (a–c) indicate significant differences (*p* < 0.05) for the Nagaimo N-10 sample collected at different digestion stages. Different uppercase letters (A–C) indicate significant differences (*p* < 0.05) for the Nebaristar sample collected at different digestion stages.

Protein digestibility (%), the SDS-PAGE profile and the free amino acids profile of the two yam tuber samples were examined to evaluate the digestion characteristics of yam protein during simulated in vitro digestion. The changes in protein content of both yam samples were generally divided into three stages according to the digestion process (Figure 2). The protein digestibility of both samples was dramatically increased by 9.1% and 26.5% for Nagaimo N-10 and Nebaristar in the first 60 min of SGD, respectively. Subsequently, the protein digestibility remained stable from G60 to G120. Then, statistically significant (*p* < 0.05) increases in protein digestibility were observed during the following SID stage, reaching 42.9% and 49.4% for Nagaimo N-10 and Nebaristar, respectively (Figure 2). Compared to the protein digestibility at G0, it increased 1.7- and 2.9-fold after 4 h of simulated gastrointestinal digestion. The tread of protein digestibility as observed in the current study was in agreement with the previous results on the protein digestibility of fermented soybeans (natto) using a similar gastrointestinal in vitro digestion model [12]. 

Yam tuber contains plenty of mucilage, comprising of at least nine major soluble proteins, such as dioscorin, mannan-binding lectin and others, in the N-terminal amino acid sequence [18]. At the beginning of digestion after pH adjustment (G0), the protein digestibility (17.1–24.7%, Figure 2) was attributed to the soluble proteins in the yam mucilage, which could be easily released in the yam slurry. The dramatic increase in protein digestibility from G0 to G60 indicated that the digestive enzymes played a role in digesting the proteins in yam solids. The protein digestibility remained stable from G60 to G120, which was most likely due to sufficient enzymes for the reaction with 1.97–2.48% of total yam proteins during the first 60 min of SGD. It has been reported that the yam starch granule was wrapped by or adhered to protein fragments, polysaccharides and lipids [19,20], which was also supported by the current study (Section 3.4). Due to the presence of the AMG in the SIF, the partial starch in the yam tuber was promptly hydrolysed, accelerating the release of yam proteins that were bound with the starch granules. On the other hand, the yam chyme was further digested in SIF also due to the presence of pancreatin that is equipped with proteolytic, lipolytic and amylolytic activities [21], accelerating the release and conversion of protein during SID. 

#### 3.2.2. Soluble Protein Fractions and Distribution

**Figure 3:**
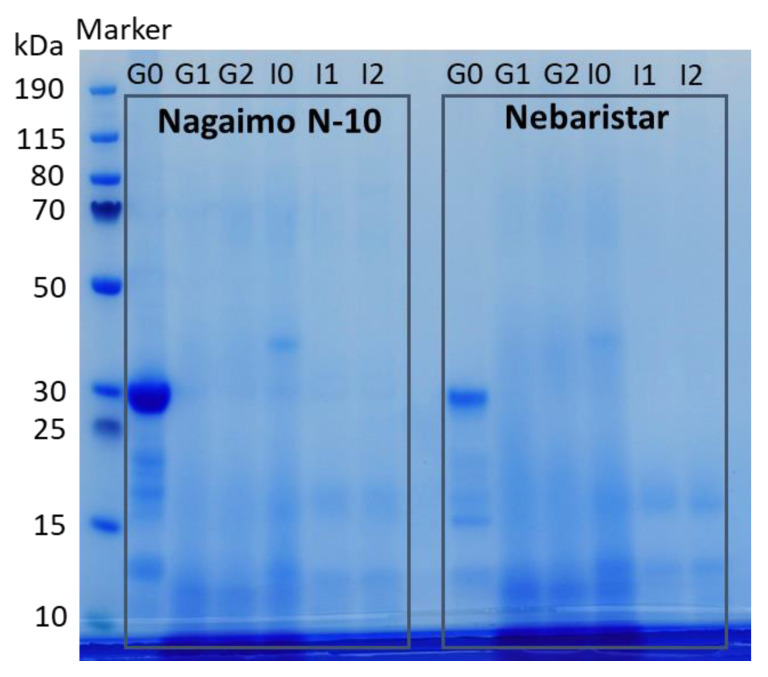
The electrophoretic patterns of the undigested yam protein and soluble protein subfractions at different digestion stages.

The electrophoretic patterns of the undigested yam protein and soluble protein subfractions at different digestion stages are shown in Figure 3. The undigested soluble yam protein at G0 showed protein bands mainly lower than 40 kDa, and this was observed in both samples (Nagaimo N-10 and Nebaristar). Among these, the most intense bands around 30 kDa (Figure 3, line G0) represented the major storage protein in the yam tuber, namely, dioscorin [4]. This result was in accordance with the previous studies conducted by Silva do Nascimento, Caju de Oliveira [7] and Nagai, Nagashima [5]. In addition, other protein bands observed with a lower MW from 13 to 22 kDa of both samples (Figure 3, line G0) might be related to the albumin proteins in yam, which was reported as having enhanced properties of protein solubility, gelling capacity and foamability [22].

The dioscorin and other proteins mentioned above almost disappeared after 60 min of SGD, mainly due to the presence of pepsin in SGF. In the meantime, the bands represented the proteins with a MW lower than 13 kDa became more intense than the initials ones at G0 with the SGD progressed (Figure 3, line G1 and G2), indicating a hydrolysis of the proteins into smaller peptide fragments in this stage. Similar results could be found in many studies related to the simulated gastrointestinal digestion of plant proteins [7,23,24]. Furthermore, Figure 3 obviously shows that the gel became clearer as the SID progressed, which means the polypeptides obtained in the SGD stage were further broken down by pancreatin into a large number of oligopeptides and amino acids with a MW below 10 kDa. The result was supported by Silva do Nascimento, Caju de Oliveira [7], who found that polypeptides with a relatively high MW were converted into oligopeptides with a MW lower than 3 kDa after complete simulated in vitro digestion. Nikoo, Regenstein [25] reported the peptide weight distribution of rainbow trout during autolysis. The results showed that 93% of peptides had low MWs of <1 kDa.

According to the electrophoretic pattern profile of yam protein during simulated in vitro digestion, it was suggested that proteins in the two raw yam samples (Nagaimo N-10 and Nebaristar) could be easily digested into low MW peptides and amino acids (refer to Section 3.2.3) during the gastrointestinal tract. Their functional groups, therefore, were exposed to be more capable of performing various bioactivities [26]. 

#### 3.2.3. Changes in Amino Acid Composition during Simulated In Vitro Digestion

The amino acid constitution is of great importance in evaluating nutritional quality, and the release of free amino acids is related to the bioaccessibility and bioavailability of food protein. The profile changes in amino acids of the yam tubers during simulated in vitro digestion are shown in Table 1. Generally, compared to the yam samples before digestion, the total amino acid contents obtained in the digestive fluid was obviously increased after digestion, with an increment of 1.4-fold for both samples. Of these, the increment of essential amino acids (EAA), hydrophobic amino acids (HBAA), hydrophilic amino acids (HLAA), aromatic amino acids (AAA) and antioxidant amino acids (AOAA) was 1.4–2.7-fold for Nagaimo N-10 and 1.3–3.7-fold for Nebaristar, respectively. It is worth mentioning that the EAA in both samples increased almost two times after in vitro digestion, implying the potential nutritional values of raw yam tuber as a great source of EAA. The EAA cannot be synthesized by the human body and must therefore come from food. EAA are vital constituents in the diet that play a role in the synthesis of protein for the human body [27]. The highest increase in the individual amino acid content was Tyr for both two samples, followed by Leu, Phe, Lys, Met, Ile and Val, indicating a high bioaccessibility of these amino acids in vitro.

Comparing the amino acid concentrations between Nagaimo N-10 and Nebaristar (Table 1), the latter sample showed significantly (*p* < 0.05) higher values in EAA, HLAA, AAA and AOAA before in vitro digestion than the former, ranging from 1.2 to 2.3 times. On the other hand, the Nebaristar also showed a higher release in EAA, HLAA, AAA and AOAA after 4 h of simulated gastrointestinal digestion. The results indicated that the Nebaristar could be a better choice according to the amino acid profile for consumers when purchasing yam tubers. Notably, Nebaristar contains 2.3 times more Arg than that in Nagaimo N-10, which was reported as a conditional essential amino acid for adults, with the functions of reproductive, cardiovascular and immune improvement [28]. The concentration and bioavailability of amino acids in the intestine could impact the protein metabolism at the splanchnic and peripheral tissues [29]. The current finding could serve as a nutritional reference to customers when buying yam tubers for the purpose of being eaten raw.

### 3.3. Changes in Total Antioxidant Capacity and Total Phenolic Content during Simulated In Vitro Digestion

**Figure 4:**
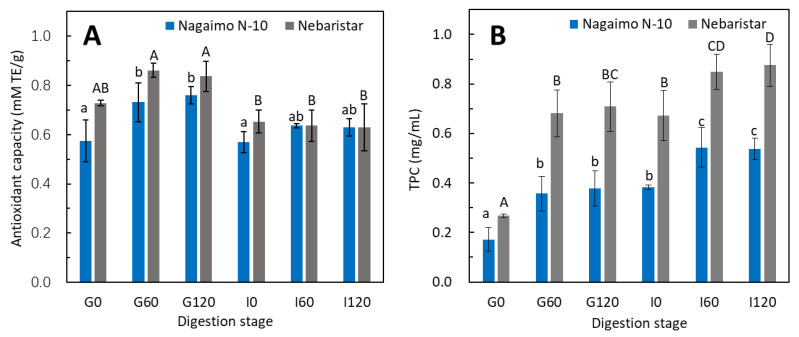
Changes in total antioxidant capacity (TAC) and total phenolic content (TPC) of the raw yam tubers during simulated in vitro digestion. Different lowercase letters (a–c) indicate significant differences (*p* < 0.05) for the Nagaimo N-10 sample collected at different digestion stages. Different uppercase letters (A–D) indicate significant differences (*p* < 0.05) for the Nebaristar sample collected at different digestion stages. (**A**): Changes in total antioxidant capacity (TAC) of both samples; (**B**): Changes in total phenolic content (TPC) of both samples.

The total antioxidant capacity (TAC) and total phenolic content (TPC) are usually used to evaluate the bioactivity of vegetables and fruits [15]. Thus, the TAC and TPC were selected to evaluate the bioactive functionality of the raw yam tubers before and after simulated in vitro digestion. Both samples showed a similar trend of changes in TAC and TPC (Figure 4), in which the TAC increased by 18–27% after the SGD and decreased by 22–25% after adjusting the pH to 6.8 (I0), as well as a relatively constant radical scavenger activity in the 2 h of SI stage. Comparing the changes in the TAC between the two samples (Figure 4A), Nebaristar exhibited a higher TAC than the Nagaimo N-10 before in vitro digestion commenced, indicating a better performance in radical scavenger activity. This trend of the TAC changes as observed in the current study is in agreement with the result obtained by Ketnawa, Suwannachot [30], who studied the changes in the antioxidant potential of crisphead lettuce during in vitro gastrointestinal digestion. The increase in the TAC during SGD was most likely due to protein hydrolysis into short-chain peptides and amino acids by the pepsin contained in the SG fluid and the pH changes. The protein hydrolysates are capable of aggregating during hydrolysis, and a higher peptide concentration resulted in a stronger antioxidant capacity [31]. On the other hand, the increase in the antioxidant capacity of polyphenols during simulated gastrointestinal digestion was due to the deprotonation of the hydroxyl moieties present on the aromatic rings of the phenolic compounds [32]. The environmental transition from stomach to intestine may lead to structural changes in phenolic molecules, which was probably due to the ionisation of the hydroxyl groups. The conversion in pH has been known to influence the racemisation of molecules, which could cause the changes in their biological reactivity; in this regard, this may urge the antioxidant factors to be more active in the early stage of the digestion process, as racemisation can increase with the rise in pH in other compounds [33]. On the other hand, the increase in pH from 2.0 to 6.8 of the digestive fluid promoted the formation of the protein–phenolic complex that could reduce or mask the antioxidant capacity [34].

Similarly, a significant (*p* < 0.05) increase in the TPC of both samples was observed, from 0.17–0.27 (G0) to 0.38–0.71 (G120) and 0.54–0.88 mg (I120) GAE/g during in vitro digestion (Figure 4B). Comparing the two samples, the TPC in the digestive fluid of the Nebaristar was significantly higher than that of the Nagaimo N-10 through 4 h of digestion, indicating a higher bioaccessbility in phenolic compounds of Nebaristar. This means that the Nebaristar could be a better option for the customer who expects to acquire more polyphenols when buying yams. This result was supported by previous studies on the changes in TPC of 23 commercially available vegetable juices during in vitro digestion using a similar evaluation method [35] and changes in the polyphenols of Tamarillo yoghurts [29]. During in vitro digestion, the protein and starch of the yam tuber were hydrolysed (Figure 1, Figure 2 and Figure 3) by the hydrolytic enzymes and pH changes, resulting in the release of polyphenols and an increase in the TPC. However, the TPC is not only dependent on the polyphenol content but also on the structure and interactions among polyphenols. In addition, as plenty of compounds hinder the Folin–Ciocalteu assay, this is therefore an approximate method to determine the TPC. It is worth noting that the tendency of TPC was not in accordance with that of the TAC during SID. This phenomenon could be due to the continuous release of polyphenols during SID masking the impacts of the protein–phenolic complex on the TPC, leading to a continuous increase in the TPC during in vitro digestion.

### 3.4. Changes in Microstructures of Yam Tubers during Simulated In Vitro Digestion

**Figure 5:**
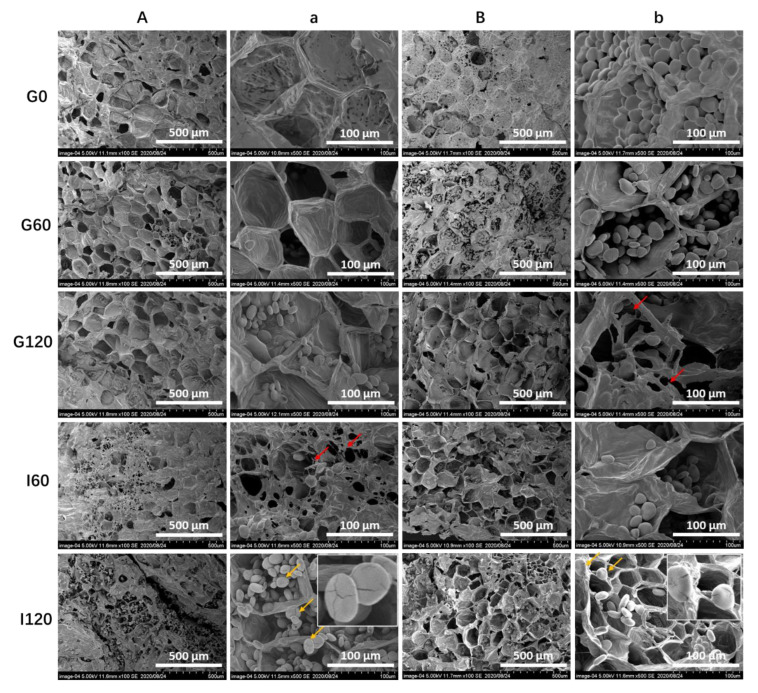
SEM micrographs showing microstructures of raw yam tubers ((**A**,**a**): Nagaimo N-10; (**B**,**b**): Nebaristar) during in vitro digestion. All the samples with the same raw letter were collected at the same time during digestion (G0/G60/G120: 0, 60 and 120 min during simulated gastric digestion; I60/I120: 60 and 120 min during simulated intestinal digestion). The micrographs for the (**A**,**B**) columns show the microstructure of samples at the magnification of 100×, and the (**a**,*b*) columns indicate the microstructures at 500× magnification.

To better understand the mechanism responsible for the starch and protein hydrolysis and the changes in TAC and TPC, the microstructure of the two yam samples at each digestion stage (G0, G60, G120, I60 and I120) was studied (Figure 5). The raw yam tuber samples showed a compact and honeycomb-like structure before digestion, and a large number of oval and spherical starch granules entrapped in the parenchyma cellular compartment were obviously observed (Figure 5, line G0). This finding was supported by previous studies on the structures of other starchy tuber plants such as potato and sweet potato [36,37]. Few differences in microstructure between the two yam samples were observed before and after digestion.

Some visible cellular breakage can be seen with the digestion progressed (Figure 5, marked with red arrows), resulting in more starch granules and protein molecules being leaked and exposed to the digestive fluid. This phenomenon was intensified during the SID stage, showing that more cell structures were broken to some extent. However, the parenchyma cellular compartments were mostly kept intact to the end of 4 h in vitro digestion (Figure 5, line G120). Notably, a mass of starch granules can be seen in the parenchyma cellular compartments, and some of them clearly showed visible cracks (Figure 5, marked with yellow arrows) on the surface. The large amount of starch remining after digestion confirmed the low starch hydrolysis (%), as discussed in Section 3.1. In addition, the result obtained in the current study was apparently different from those of cooked yam tubers (>70% for the samples cooked by boiling and steaming), which showed relatively high starch hydrolysis after digestion.

## 4. Conclusions

This work studied the simulated in vitro digestive characteristics of two typical raw yam tubers in the Japanese diet, focusing on the changes in protein profile, protein and starch digestibility, antioxidant capacity and microstructure. In sum, results showed that the raw yam tuber is a desirable food considering its low starch digestibility and potential slow glycemic increase after consumption. Nebaristar could be a more nutritional yam tuber variety than the Nagaimo N-10 when being eaten raw, according to the higher protein digestibility and free amino acid content, as well a the higher bioaccessibility in the EAA, HBAA, AOAA and TPC. This research creates an insight into the digestive characteristics of yam tubers, promoting the realization and popularization of the consumption of yam tubers as part of a healthy diet.

## Figures and Tables

**Table 1:** Release of free amino acids in yam tubers during simulated in vitro digestion.

	Nagaimo N-10			Nebaristar		
Amino Acid	Before Digestion	After Digestion	Increment (Fold)	Before Digestion	After Digestion	Increment (Fold)
	nmol/g Fresh Yam			nmol/g Fresh Yam		
Asp	465.2 ± 8.1	599.9 ± 14.7	1.3	669.3 ± 4.9	802.2 ± 29.3	1.2
Thr	770.7 ± 16.8	957.4 ± 20.1	1.2	1374.7 ± 82.8	1443.7 ± 18.9	1.1
Ser	4941.8 ± 92.2	5485.8 ± 135.4	1.1	5881.7 ± 181.8	6218.2 ± 78.8	1.1
Glu	420.4 ± 0.6	624.7 ± 17.7	1.5	496.3 ± 15.7	608.4 ± 40.6	1.2
Gly	1069.7 ± 14.4	1273.4 ± 7.3	1.2	984.0 ± 10.7	1131.5 ± 15.2	1.1
Ala	4797.5 ± 60.2	5426.4 ± 68.6	1.1	3255.0 ± 34.9	3647.4 ± 48.0	1.1
Val	753.2 ± 12.6	1114.1 ± 11.0	1.5	824.5 ± 11.6	1243.4 ± 17.7	1.5
Met	177.7 ± 0.9	341.5 ± 3.1	1.9	242.5 ± 3.3	495.3 ± 20.1	2.0
Ile	473.3 ± 3.7	806.5 ± 9.8	1.7	611.3 ± 7.3	1089.2 ± 17.1	1.8
Leu	548.8 ± 4.2	1607.8 ± 23.6	2.9	848.7 ± 10.8	2920.7 ± 36.9	3.4
Tyr	115.6 ± 1.2	685.6 ± 10.1	5.9	139.8 ± 1.8	1211.5 ± 15.8	8.7
Phe	427.3 ± 3.6	1170.4 ± 6.7	2.7	484.8 ± 5.6	1964.9 ± 7.8	4.1
His	253.5 ± 1.1	333.9 ± 3.2	1.3	345.4 ± 6.1	438.2 ± 9.9	1.3
Lys	384.1 ± 2.1	966.8 ± 13.8	2.5	755.1 ± 7.5	1770.5 ± 16.8	2.3
Arg	3257.0 ± 18.5	4253.6 ± 56.6	1.3	8631.8 ± 118.2	10,317.1 ± 131.6	1.2
Pro	102.2 ± 6.8	123.7 ± 1.6	1.2	225.1 ± 2.8	241.1 ± 16.3	1.1
Cysta *	109.5 ± 0.0	148.8 ± 4.4	1.4	252.6 ± 3.3	315.9 ± 1.3	1.3
In total	19,067.5 ± 247.1	25,920.4 ± 407.7	1.4	26,022.6 ± 509.5	35,859.2 ± 522.1	1.4
EAA	3788.7 ± 41.4	7298.6 ± 84.7	1.9	5487.0 ± 129.8	11,366.0 ± 137.4	2.1
HBAA	7505.0 ± 99.9	11,424.9 ± 139.0	1.5	6884.3 ± 81.8	13,129.5 ± 180.9	1.9
HLAA	4780.3 ± 30.5	6778.9 ± 105.9	1.4	10,897.9 ± 152.3	13,936.4 ± 228.3	1.3
AAA	796.5 ± 5.9	2190.0 ± 20.1	2.7	970.0 ± 13.6	3614.7 ± 33.6	3.7
AOAA	1185.8 ± 13.5	2803.9 ± 29.3	2.4	1690.2 ± 23.0	4667.0 ± 71.3	2.8

Asp = aspartic acid, Thr = threonine, Ser = serine, Glu = glutamic acid, Gly = glycine, Ala = alanine, Val = valine, Met = methionine, Ile = isoleucine, Leu = leucine, Tyr = tyrosine, Phe = phenylalanine, His = histidine, Lys = lysine, Arg = arginine, Pro = proline, Cysta = cystathionine; EAA = essential amino acids: His, Ile, Leu, Lys, Met, Phe, Thr and Val; HBAA = hydrophobic amino acids: Ala, Val, Ile, Leu, Tyr, Phe, Trp, Pro, Met and Cys; HLAA = hydrophilic amino acids: Arg, Asp, His, Lys, Glu; AAA = aromatic amino acids: Phe, Trp, Tyr and His; AOAA = antioxidant amino acids: Trp, Tyr, Met, Cys, His, Phe and Pro; * cysteine was determined in the form of cysta; tryptophan (Trp) was not reported, since it is unstable and produces ammonia, and thus the amino acid standard mixture was prepared in the absence of the Trp.

## Data Availability

Data is contained within the article.

## Appendix A

**Table A1:** Moisture content, crude protein and total starch of the two samples.

	Nagaimo N-10	Nebaristar
Moisture content (%)	82.2 ± 1.2	78.0 ± 2.4
Crude protein (dry basis %)	11.1 ± 0.2	11.3 ± 0.3
Total starch (dry basis %)	67.3 ± 1.6	72.4 ± 0.7
